# Post-Surgical Ablative or Adjuvant Radioiodine Therapy Has No Impact on Outcome in 1–4 cm Differentiated Thyroid Cancers without Extrathyroidal Extension

**DOI:** 10.3390/jcm10194452

**Published:** 2021-09-28

**Authors:** Simone De Leo, Matteo Trevisan, Carla Colombo, Giacomo Gazzano, Sonia Palazzo, Leonardo Vicentini, Luca Persani, Laura Fugazzola

**Affiliations:** 1 Division of Endocrine and Metabolic Diseases, Istituto Auxologico Italiano IRCCS, 20149 Milan, Italy; simonedeleo86@gmail.com (S.D.L.); carla.colombo1@unimi.it (C.C.); luca.persani@unimi.it (L.P.); 2 Department of Pathophysiology and Transplantation, University of Milan, 20149 Milan, Italy; matteo.trevisan@unimi.it; 3 Pathology Unit, Istituto Auxologico Italiano IRCCS, 20149 Milan, Italy; g.gazzano@auxologico.it (G.G.); s.palazzo@auxologico.it (S.P.); 4 Endocrine Surgery Unit, Istituto Auxologico Italiano IRCCS, 20149 Milan, Italy; viceleo@hotmail.com; 5 Department of Medical Biotechnologies and Translational Medicine, University of Milan, 20149 Milan, Italy

**Keywords:** radioiodine ablation, differentiated thyroid cancer, cancer remission, cancer recurrence risk

## Abstract

Whether to conduct remnant ablation or adjuvant radioactive iodine (RAI) therapy in patients with intrathyroidal differentiated thyroid carcinoma (DTC), sized 1.1–4 cm, is debated. We evaluated the impact of RAI on outcome in this category of DTCs. We retrospectively enrolled 308 patients submitted to total thyroidectomy: 198 had tumors sized 1.1–2 cm (Group 1) and 110 of 2.1–4 cm (Group 2). Both groups were divided into patients receiving and not receiving RAI after surgery. RAI+ and RAI− patients did not significantly differ, regarding several clinical and pathological features. Final outcome was defined according to dynamic risk stratification. Remission was observed in the majority of Group 1 and Group 2 patients and outcome did not significantly differ between RAI+ and RAI− patients: respectively, 95.8% vs. 93.7% in Group 1, and 87.7% vs. 86.5% in Group 2. The majority of persistent cases, either RAI+ or RAI−, received therapeutic RAI administration, and about 50% of RAI− cases had an excellent response at final follow up, whereas no RAI+ persistent patients had a beneficial effect. Our findings demonstrate that patients with an intrathyroidal DTC sized 1.1–4 cm do not benefit from RAI. The outcome of these patients remains favorable, and the few patients with persistent diseases can be treated with RAI during follow up.

## 1. Introduction

Differentiated thyroid cancer (DTC) accounts for more than 90% of thyroid tumors and its incidence has progressively increased in the last few decades [1]. Prognosis is generally favorable, and mortality, albeit slightly increasing in the recent years, is generally low [1]. For this reason, attention is mainly focused on the risk of recurrence, reliably predicted by the American Thyroid Association (ATA) risk classification [2]. In particular, up to 20% of DTC will have a recurrence, though this rate reduces to 5% in ATA low-risk patients [3]. Radioactive iodine (RAI) has been a standard of care for patients with DTC, but its role in the lower risk categories has been questioned because of the indolent behavior of these tumors. RAI may be used with different goals: (i) to ablate remnant thyroid tissue, (ii) as adjuvant treatment, in order to destroy suspected but not identified remaining disease, and (iii) as a treatment of a known disease [4]. After surgery, either remnant ablation or adjuvant treatment can be administered. Remnant ablation has a particular role in determining initial staging and facilitating follow up, while adjuvant treatment additionally improves disease-specific and progression-free survival, decreases recurrences, and has a curative intent, even though in many patients, surgery may be sufficient [4]. While both European and American Thyroid and Nuclear Medicine Societies agree not to perform RAI ablation in microcarcinomas (≤1 cm) [2,5,6,7], there is still controversy regarding low-risk thyroid tumors >1 cm. ATA guidelines state that DTC between 1 and 4 cm should not routinely be treated by RAI [2], while the Nuclear Medicine Societies recommend RAI ablation for all tumors larger than 1 cm [6,7], based on the impossibility to reliably discriminate between patients who could benefit or not from this treatment [8]. The aim of our study was to evaluate (i.e., without minimal or gross extrathyroidal extension and without local and/or distant metastases) the impact on outcome of radioiodine used for ablative or adjuvant purposes in patients with intrathyroidal DTCs sized 1.1–4 cm.

## 2. Materials and Methods

### 2.1. Patients

This is a monocentric study, and we included all consecutive patients followed up in our academic reference center from January 1990 to November 2020 and for whom pathological and clinical data were available. Full information was recorded at each visit and retrospectively analyzed. From a cohort of more than 1500 patients, we only selected patients who were submitted to total thyroidectomy with a pathological diagnosis of DTC, whose tumor size was between 1.1 and 4.0 cm, without lymph node or distant metastases and without extrathyroidal extension. The patients included were divided into 2 groups, one including cases with the largest tumor size between 1.1 and 2.0 cm (Group 1, *n* = 198), and another including those with the largest tumor size between 2.1 and 4.0 cm (Group 2, *n* = 110). Both cohorts were subdivided in two groups: one group with patients who performed RAI after surgery, i.e., for remnant ablation or adjuvant treatment (RAI+, *n* = 71 Group 1 and *n* = 73 Group 2) and the other whose patients did not perform RAI after surgery (RAI−, *n* = 127 Group 1 and *n* = 37 Group 2). In the period 1990–2020, different indications were available to the scientific community related to the management of thyroid cancer patients. In particular, until 2006, we followed American guidelines [9], while starting from 2006, we followed the European Consensus for the management of differentiated thyroid cancer [5]. It is worth noting that in both guidelines, RAI ablation was not routinely recommended, only for low-risk or unifocal tumors ≤1 cm. On the other hand, ATA guidelines on the management of DTC published in 2009 reported that RAI ablation was definitely recommended only for tumors >4 cm or for tumors of any size with a gross extrathyroidal extension [10]. Upon this publication, we decided not to submit to RAI ablation all intrathyroidal DTCs ≤4 cm, independently from post-operative thyroglobulin (Tg) levels. Consistently, though the dates for surgery are widely scattered in the whole series, the mean year of intervention for RAI+ patients was 2005 in both groups, while it was 2011 and 2010 for RAI− cases of Group 1 and 2, respectively. Importantly, all the patients included have been operated by the same expert endocrine surgeon (LV). The study was performed in accordance with the ethical standards of the Institutional Research Committee and with the 1964 Helsinki Declaration. All patients provided informed consent to the use of their anonymized clinical data for research purposes, at diagnosis or during follow up. Patients are included in a protocol related to the management of the clinical and genetic data of thyroid cancer patients approved by the Ethical Committee of the Istituto Auxologico Italiano (#05C825_2018).

### 2.2. Follow up, Biochemical Assessment of Serum Thyroglobulin (Tg) and Antithyroglobulin Antibodies (TgAb), and Neck Ultrasound

The first evaluation was performed at discharge from the Surgery Unit and after two weeks at the time of pathology report availability. Afterwards, patients were evaluated three months after surgery and then every 6 or 12 months according to clinical response to treatment. Patients treated with RAI were also evaluated 2 months after RAI, too. RAI ablation/adjuvant treatment was administered after a mean of 4.3 ± 3 months (median 4, range 1–12 months) after surgery. At each follow up visit, Tg and thyroglobulin antibodies (TgAb) were measured and neck ultrasound examination was made by the same operator. Other imaging studies (e.g., computed tomography, magnetic resonance imaging, fluorodeoxyglucose (FDG)-positron emission tomography scans) were performed when appropriate. Starting from early 2016, only basal Tg was dosed with an ultrasensitive assay (Elecsys Tg II-Roche Diagnostics, Basilea, Switzerland, analytical sensitivity 0.04 mcg/L), while previously recombinant human TSH (rhTSH)-stimulated Tg was evaluated along with basal levels. For thyroglobulin antibodies, Elecsys^®^ anti-Tg Roche Diagnostics (upper limit normal 115 kU/L) was used.

### 2.3. Dynamic Risk Stratification

According to ATA guidelines, response to treatment was defined by dynamic risk stratification (DRS). Patients submitted to total thyroidectomy and RAI ablation had excellent response—i.e., disease remission—if they had negative imaging and either suppressed Tg < 0.2 ng/mL or stimulated Tg < 1 ng/mL; biochemically incomplete response if negative imaging and suppressed Tg ≥ 1 ng/mL, or stimulated Tg ≥ 10 ng/mL or rising AbTg levels; structurally incomplete response if structural or functional evidence of disease; indeterminate response if lack of previous criteria [2]. In patients treated only by surgery, response to treatment was defined following the DRS criteria proposed by Momesso et al. [11]. Therefore, excellent response was defined by non-stimulated Tg < 0.2 ng/mL (or stimulated Tg < 2 ng/mL), undetectable TgAb and negative imaging. On the other hand, tumor persistence and/or recurrence was defined by either biochemical (i.e., non-stimulated Tg > 5 ng/mL, or stimulated Tg > 10 ng/mL, or increasing Tg/AbTg values and negative imaging) or structural (positive imaging or functional test regardless of Tg/AbTg level) response. In case of uncertain imaging findings, non-stimulated Tg 0.2–5 ng/mL (or stimulated Tg 2–10 ng/mL), or stable or declining TgAb levels, response to therapy was considered to be indeterminate.

### 2.4. Statistical Analysis

Descriptive analysis of both quantitative and qualitative data has been performed. Statistical differences between continuous variables have been defined by means of the Mann–Whitney *U* test, whereas differences between discrete variables have been appraised with *χ*2 test and Fisher’s Exact Test. Statistical significance has been defined as *p* < 0.05. All statistical analyses have been performed using MedCalc Statistical Software version 19.2.0 (MedCalc Software bvba, Ostend, Belgium).

## 3. Results

### 3.1. Patient Cohort Characteristics

In both groups, no significant differences were found in the clinical characteristics (patient gender, mean diameter, histotype, histological variants, multifocality, associated thyroiditis, vascular invasion, ATA risk) among RAI+ and RAI− cases, with the exception of age at diagnosis and the duration of follow up (Table 1). In particular, in both groups, age was significantly higher in RAI− patients (40.4 ± 14.2 vs. 49.9 ± 13.3 years, *p* < 0.001 in Group 1 and 41.5 ± 14.2 vs. 47.3 ± 14.2 years, *p* = 0.018 in Group 2). Moreover, RAI+ had a follow up longer than RAI− patients (115.4 ± 69.4 vs. 61.9 ± 72.3 months, *p* < 0.001 in Group 1 and 113 ± 86.4 vs. 84.8 ± 128.2 months, *p* < 0.001 in Group 2). The overall median follow up was 67 months.

RAI+ patients of Group 1 received a mean activity of 2834.2 MBq (range 370–5550, median 3145 MBq), 11 (15.5%) after rhTSH stimulation and the remaining 60 (85.5%) after TSH withdrawal; RAI+ patients of Group 2 received a mean activity of 2963.7 MBq (range 555–5550, median 3367 MBq), 26 (35.6%) after rhTSH stimulation and the remaining 47 (64.4%) after TSH withdrawal.

### 3.2. Outcome after Initial Treatment

**Figure 1:**
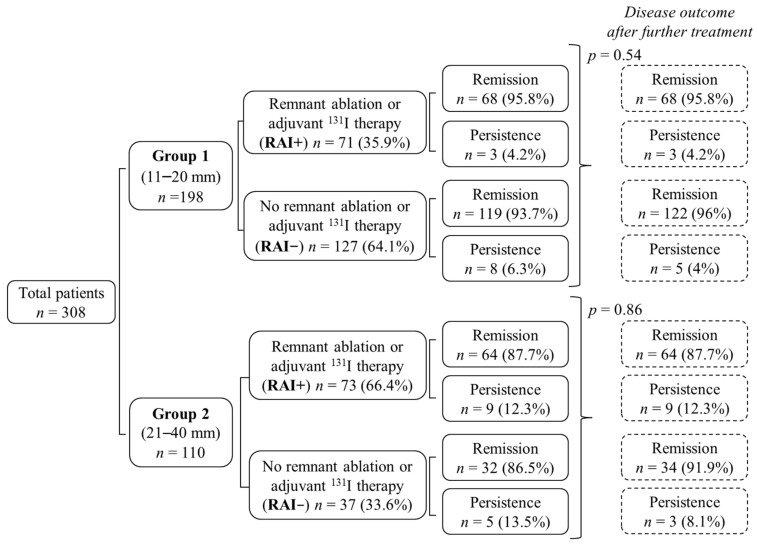
Outcome at the last follow up visit of patients of Group 1 (11–20 mm) and Group 2 (21–40 mm). After surgery, patients were submitted to radioiodine ablative/adjuvant treatment (RAI+) or not (RAI−). The outcome after eventual additional treatment of persistent cases is also shown (see Table 2 for details).

In both groups, there was no significant difference in the remission rate among RAI+ and RAI− cases (*p* = 0.54 in Group 1 and *p* = 0.86 in Group 2) (Figure 1).

In particular, remission was observed in the majority of both RAI+ patients (95.8% in Group 1 and 87.7% in Group 2) and RAI− cases (93.7% of Group 1 cases and 86.5% of Group 2). No significant difference was found in the remission rate, neither between RAI+ cases of Groups 1 and 2 (*p* = 0.08) nor between RAI− patients of Groups 1 and 2 (*p* = 0.15).

### 3.3. Final Outcome of Persistent Cases

A total of 25/308 (8.1%) persistent cases were observed (11 in Group 1 and 14 in Group 2) (Table 2).

The rate of persistence, being in almost all cases biochemical or loco regional, was slightly higher, though not statistically significant, in RAI− cases (Figure 1). Seventeen out of 25 persistent cases (9 in Group 1 and 8 in Group 2) underwent an additional treatment, mainly RAI administration. It is worth noting that in RAI− persistent cases, the mean time after surgery for a therapeutic treatment was 12.6 ± 9.2 months (median 10, range 5–34 months) (Table 2). In Group 1, 4/9 patients received further treatment for a biochemical and 5/9 for a structural persistence, while, in Group 2, 4/8 received treatment for a biochemical and 4/8 for a structural persistence. At the final follow up, among the 11 persistent patients of Group 1, the 3 RAI+ cases had a biochemically or structurally incomplete response, despite 2 of them receiving an additional ^131^I treatment. Seven out of eight RAI− cases were treated with ^131^I and 3/7 of them had an excellent response, while the others are still in persistence. Among the 14 persistent patients of Group 2, all 9 RAI+ cases were still in persistence at the final follow up (mainly biochemically or structurally incomplete response), despite additional ^131^I administration in 3 of them. Four out of five RAI− cases received radioiodine treatment and two of them had an excellent response, while the remaining are still in persistence. In summary, in both groups, no RAI+ persistent patients who received an additional ^131^I dose had a beneficial effect in terms of final response, i.e., all had a persistent disease. On the contrary, 3/8 RAI−persistent patients of Group 1 and 2/5 RAI− cases of Group 2 had an excellent response at final follow up after RAI administration (Table 2). Thus, after additional treatment, the persistence/remission rate was highly comparable between RAI+ and RAI− cases in both groups (Figure 1).

## 4. Discussion

This study indicates no benefit of ablative/adjuvant RAI therapy on the remission rate of patients with intrathyroidal DTCs sized 1.1–4 cm. We retrospectively evaluated consecutive patients, with a median follow up of 67 months, by dividing them into two groups, which were highly comparable in terms of clinical features: Group 1, including 11–20 mm tumors and Group 2, including 21–40 mm cases. Both groups were further divided into radioiodine-treated (RAI+) and not treated (RAI−). The rate of remission was >95% in Group 1 and >85% in Group 2; this was not statistically different among RAI+ and RAI− patients. The high remission rates in both RAI+ and RAI− cases are comparable to those previously reported in the literature for low-risk tumors [12,13,14]. Nevertheless, discrepancies still exist in the management of these patients, particularly when the tumor size is >1 cm, as emerging from European and American guidelines from either Nuclear Medicine or Endocrinology Societies [2,5,6,7]. This is likely due to the discordant results published so far on this topic, with some studies reporting a benefit of RAI treatment even in very-low-risk tumors, while others describing no advantages [15]. Interestingly, the impact of RAI treatment was evaluated in low-risk tumors series, often including a high proportion of microcarcinomas, with unclear or variable inclusion/exclusion criteria [16,17]. This was underlined by Sacks et al. [16], who highlighted the variable definitions of low-risk patients, making a clear comparison among studies difficult. Because general agreement exists about the lack of indication for RAI ablation in microcarcinomas, we excluded those cases from our series and analyzed, to our knowledge, for the first time, only larger intrathyroidal tumors, sized 11–40 mm. In this study, the persistence rate ranged 4–6% in Group 1 and 12–14% in Group 2, without significant differences between RAI+ and RAI− cases. An additional treatment after surgery (mainly RAI administration) was carried out in 11 out of the 13 RAI− persistent patients, after a mean time-lapse of 12.6 ± 9.2 months (median 10 months), and about half of them had an excellent response to treatment. Thus, our data confirm that the delayed RAI administration has no impact on the final outcome [18,19] and indicate that, in patients with a tumor size up to 4 cm, RAI treatment can be safely planned during the follow up, even several months after surgery. On the other hand, a therapeutic ^131^I dose was administered in 5/12 persistent RAI+ patients, without any improvement in terms of DRS. Since persistent cases were due, in all cases (with the exception of one patient with distant metastases), to a biochemically or locoregionally structural disease, our data confirm those from a previous report on the limited benefit of therapeutic RAI administration in these cases [20]. It is worth noting that, at the final follow up, the persistence/remission rate was superimposable in RAI+ and RAI− cases of both groups.

Our study, besides its retrospective nature, has some potential drawbacks. The number of RAI− patients of Group 2 is limited, thus lowering the power of the statistical analyses. Nevertheless, despite our large series, the number of thyroid tumors measuring 11–40 mm, without lymph nodal metastases and extrathyroidal extension, is infrequent. To the best of our knowledge, this remains the highest number of patients with these characteristics not submitted to RAI ablation and with an available follow up. Moreover, in Group 1, age at diagnosis was significantly lower in RAI+ cases, but this factor has just a strengthening value for the RAI+ group since younger are predicted to have a better outcome than older patients [21]. RAI− patients had a shorter follow up than RAI+ cases, because we decided not to RAI ablate these cases only after 2009, upon ATA indications for a selective use of ablation treatment. Nevertheless, since the majority of recurrences are predicted to be diagnosed within the first 5 years of follow up [22,23], even our shorter mean follow up (62 months) is expected to be long enough to diagnose possible relapses.

Finally, patients with microscopic extrathyroidal extension (mETE) were not included in the study. In 2017, the American Joint Committee on Cancer (AJCC) published the eighth Edition of the AJCC/TNM (tumor-node-metastasis) cancer staging system and downgraded these tumors that were previously considered T3 into T1 or T2 according to their tumor size [24]. In accordance with our findings, a recent study suggested that RAI treatment could be avoided or postponed in these cases, though 40% of cases had RAI ablation during the follow up because of detectable thyroglobulin levels [25]. Prospective studies trying to compare outcome of patients with mETE treated or not treated by adjuvant RAI therapy are needed to shed light on these patients. Still, we believe that selective use of RAI in these patients is also warranted. Limiting RAI use, in inappropriate or unneeded situations, can provide a benefit not only from a health point of view but also on an economic basis: a cost of $10–15 million per year has been estimated in the United States for the unnecessary treatment of micropapillary thyroid carcinoma [26].

## 5. Conclusions

In conclusion, our study reports no benefit of ablative/adjuvant RAI therapy in all DTC tumors of 1–4 cm and without extrathyroidal extension. The rate of persistence, mainly biochemical or locoregional, is low in this category of patients, and independent of the administration of RAI ablative or adjuvant treatment. The outcome at the final follow up is extremely favorable and there is no difference in terms of rate and site of recurrence if RAI treatment is postponed in the infrequent persistent cases.

## Figures and Tables

**Table 1:** Baseline histopathological and clinical characteristics of the 2 groups of patients.

Characteristics		Group 1 (11–20 mm)			21–40 mm		
		RAI+	RAI−	*p*	RAI+	RAI−	*p*
Age at diagnosis (year)	Mean	40.4 ± 14.2	49.9 ± 13.3	<0.001	41.5 ± 14.2	47.3 ± 14.2	0.018
	Range	17–81	20–80		18–79	7–78	
Gender, *n* (%)	Female	61 (85.9)	100 (78.7)	0.21	59 (80.8)	28 (75.7)	0.53
Size, cm	Mean	1.52 ± 0.29	1.48 ± 0.27	0.38	2.84 ± 0.53	2.8 ± 0.56	0.91
	Range	1.1–2	1.1–2		2.1–4	2.1–4	
Histotype, *n* (%)	Papillary	64 (90.1)	116 (91.3)	0.78	56 (76.7)	25 (67.6)	0.31
	Follicular	7 (9.9)	11 (8.7)		17 (23.3)	12 (32.4)	
Papillary variant, *n* (%)	Classic	44 (63.8)	81 (68)	0.78	34 (60.7)	12 (42.9)	0.47
	Follicular	19 (27.5)	29 (24.4)		17 (30.4)	13 (46.4)	
	Aggressive *	2 (2.9)	5 (4.2)		2 (3.6)	1 (3.6)	
	Other **	4 (5.8)	4 (3.4)		3 (5.3)	2 (7.1)	
Multifocality, *n* (%)		26 (36.6)	38 (29.9)	0.34	21 (28)	9 (24.3)	0.62
Associated thyroiditis, *n* (%)		17 (23.9)	37 (29.1)	0.43	20 (27.4)	8 (21.6)	0.51
Vascular invasion, *n* (%)		1 (1.4)	3 (2.4)	0.65	0 (0)	1 (2.7)	0.16
ATA risk, *n* (%)	Low	68 (95.8)	119 (93.7)	0.54	71 (97.2)	35 (94.6)	0.48
	Intermediate	3 (4.2)	8 (6.3)		2 (2.8)	2 (5.4)	
Follow up, months (mean ± SD)		115.4 ± 69.4	61.9 ± 72.3	<0.001	113 ± 86.4	84.8 ± 128.2	<0.001

Legend: RAI+: patients treated with radioactive iodine (RAI) for remnant ablation or adjuvant therapy; RAI−: patients not treated with radioactive iodine (RAI) for remnant ablation or adjuvant therapy; * aggressive variants are tall cell, columnar cell, hobnail variant, and sclerosing variants; ** other variants include clear cell, oxyphil, solid, cystic, encapsulated.

**Table 2:** Clinical data and follow up of patients (Group 1 and Group 2) with persistent disease after surgery alone or after surgery and ablative/adjuvant ^131^I therapy.

Tumor Size (mm)	Istotype	First ^131^I Activity (Remnant Ablation or Adjuvant)	Further ^131^I Activity ^#^ (Therapy) (Months after Surgery)	Reason for Further Therapy	Tg-AbTg at Last Follow up (mcg/L-U/L)	DRS Response
11–20 (Group 1)	tcPTC	1110	no	-	0.04–658.0	Biochemical incomplete
	sPTC	3700	1850 (40)	Biochemical incomplete	26.0-neg.	Biochemical incomplete
	cPTC	1850	3700 (32)	Structural incomplete (CC LFN)	105-neg.	Structural incomplete (CC LFN)
	cPTC	no	3700 (4)	Biochemical incomplete	0.87-neg.	Indeterminate
	cPTC	no	3700 (25)	Structural incomplete (CC LFN)	0.04-neg.	Structural incomplete (CC + LC LFN)
	cPTC	no	3700 (34)	Structural incomplete (CC LFN)	0.2–33.6	Structural incomplete (CC LFN)
	cPTC	no	3700 (8)	Biochemical incomplete	0.31-neg.	Structural incomplete (CC LFN + Lung)
	cPTC	no	no	-	0.44–853	Structural incomplete (LC LFN)
	cPTC	no	3700 (5)	Structural incomplete (CC LFN)	0.04-neg.	Excellent
	fvPTC	no	42,180 (8)	Biochemical incomplete	0.04-neg.	Excellent
	sPTC	no	3700 (22)	Structural incomplete (CC LFN)	0.04-neg.	Excellent
21–40 (Group 2)	cPTC	1850	1110 (16)	Biochemical incomplete	1.5-neg.	Biochemical incomplete
	tcPTC	3700	no	-	8.8-neg.	Biochemical incomplete
	fvPTC	1850	5550 (9)	Structural incomplete (LC LFN)	0.04–1460	Biochemical incomplete
	FTC	2960	no	-	0.27-neg.	Indeterminate
	oFTC	3700	no	-	-	Structural incomplete (LC LFN)
	oFTC	3700	no	-	515-neg.	Structural incomplete (Lung)
	cPTC	3700	12,950 (8)	Structural incomplete (Lung + Bone)	2658-neg.	Structural incomplete (Lung + Bone)
	oFTC	1850	no **	Structural incomplete (CC LFN)	5.99-neg.	Structural incomplete (CC LFN)
	FTC	1850	no	-	1000-neg.	Structural incomplete (distant metastases *)
	cPTC	no	3700 (13)	Biochemical incomplete	0.04–177	Indeterminate
	fvPTC	no	3700 (5)	Biochemical incomplete	4.57-neg.	Structural incomplete (CC LFN)
	miFTC	no	no	-	0.2–51.04	Indeterminate
	fvPTC	no	3700 (10)	Structural incomplete (LC LFN)	0.04-neg.	Excellent
	cPTC	no	3700 (14)	Biochemical incomplete	0.04-neg.	Excellent

Legend: ^131^I dose is expressed in MBq; cPTC: papillary thyroid cancer classic variant; tcPTC: papillary thyroid cancer tall cells variant; sPTC: papillary thyroid cancer solid variant; fvPTC: papillary thyroid cancer follicular variant; oFTC: follicular thyroid cancer oxyphilic variant; miFTC: minimally invasive follicular thyroid cancer; neg.: negative; detection limit ultrasensitive thyroglobulin (Tg): 0.04 mcg/L; normal values anti thyroglobulin autoantibodies (Tg Ab): <115 U/L; ^#^ therapeutic doses of ^131^I were always administered after appropriate L-thyroxine withdrawal; * metastases to lung, bone, liver, adrenal gland, and mediastinal lymph nodes; CC: central compartment; LC: laterocervical; LFN: metastatic lymph nodes; ** patient received surgery (central compartment lymphadenectomy) for structural persistence 97 months after thyroidectomy.

## Data Availability

The data presented in this study are available on request from the corresponding author. The data are not publicly available for ethical and privacy reasons.
